# Determining the center of a keratoconus: Comparison of different tomographic parameters and impact of disease severity

**DOI:** 10.3389/fmed.2022.968318

**Published:** 2022-09-20

**Authors:** Gernot Steinwender, Alexander Kollenc, Mehdi Shajari, Michael Sommer, Andrea Borenich, Jutta Horwath-Winter, Ewald Lindner, Nora Woltsche, Wolfgang List, Andreas Wedrich

**Affiliations:** ^1^Department of Ophthalmology, Medical University Graz, Graz, Austria; ^2^Department of Ophthalmology, Medical University Frankfurt, Frankfurt, Germany; ^3^Institute for Medical Informatics, Statistics and Documentation, Medical University Graz, Graz, Austria

**Keywords:** keratoconus, corneal tomography, cornea, customized cross-linking, ectasia

## Abstract

**Purpose:**

There exists remarkable variation in definitions for the location of the center of a keratoconus. The objective of this study was to analyze deviations between locations obtained by different tomographic maps for that purpose. Furthermore, it was investigated whether these deviations are influenced by disease severity.

**Methods:**

In 162 eyes with keratoconus, corneal tomographic maps derived by Scheimpflug technology were retrospectively analyzed to determine the cone location with 5 different methods: maximum axial curvature of the front surface (Kmax), maximum tangential curvature of the front surface (tKmax), minimum pachymetry (Pachymin), maximum elevation of the front surface (ELEF), and maximum elevation of the back surface (ELEB). Distances between the locations were calculated and tested for a correlation with keratoconus severity and distance between cone and corneal vertex.

**Results:**

Cone locations derived from the curvature maps (Kmax, tKmax) showed the lowest agreement with the locations determined by pachymetry or elevation maps. The largest distances were found between Kmax and Pachymin [Median and Interquartile range: 1.19 mm (0.87, 1.60)], Kmax and ELEB [1.12 mm (0.79, 1.41)], and Kmax and ELEF [0.97 mm (0.64, 1.27)]. Low distances (<0.5 mm) were calculated between ELEB and ELEF, and ELEB and Pachymin. All of the calculated distances between the locations showed a significant negative correlation with keratoconus severity and most of them increased significantly with a more peripheral position of the cone (*p* < 0.05).

**Conclusions:**

There was low consistency between different methods for describing the location of a keratoconus. Curvature-based determinations of the cone center (Kmax, tKmax) showed the highest deviations and should not be used for that purpose. However, the discrepancies between different cone location methods diminished with increasing disease severity and more central position of the cone.

## Introduction

Keratoconus is a localized biomechanical disorder of the cornea that changes the natural shape of the cornea into a more cone-like shape (1). Refractive consequences of ectatic protrusion and thinning of the cornea in keratoconic eyes are irregular astigmatism and myopia, reducing patients quality of life (2, 3).

There is currently no cure for the disease, although patients can be helped to compensate visual impairment with spectacles or hard contact lenses. In progressive cases of ectasia the condition can be halted with interventions such as corneal cross-linking (CXL), which increases the biomechanical strength of the cornea (4).

In the standard CXL protocol first described by Wollensak et al., the cornea is soaked with riboflavin solution before exposure to a uniform beam of ultraviolet (UV) radiation (5). In a newer approach, only the affected part of the ectatic cornea instead of the entire cornea is addressed by UV energy due to a defined intensity beam profile. Thus, such customized CXL can reduce the size of the exposed cornea and could provide the same or even better therapeutic efficacy than the standard protocol with less UV energy (6–8).

An important issue in customized CXL is to identify the location of the weakest area of the cornea (6, 9, 10). As no objective clinical quantification of the biomechanically affected area is currently available, recently published studies relied on geometrical measurements of the cornea. Some investigators located the center of the UV beam at the point of maximum elevation of the posterior corneal surface (6, 8), while others decided to center the treatment at the points of axial or tangential steepest curvature of the anterior corneal surface (10).

Beside those new therapeutic approaches also a widely used diagnostic method for describing and staging keratoconus based on Scheimpflug-derived tomographic data is based on a certain definition of the center of a keratoconic cornea. With the aim of enhancing early ectatic changes in the elevation map, Belin and Duncan determined the thinnest point as the center of the disordered corneal region and eliminated a small diameter optical zone centered around that point from the standard best-fit-sphere computation (11).

Apparently, there exists remarkable variation in definitions of the center of a keratoconus and thus, the objective of this study was to analyze deviations between locations of different tomographic parameters proposed for that purpose. Furthermore, we wanted to investigate whether these deviations are influenced by disease severity.

## Methods

In this retrospective study, corneal tomography maps of 162 right and left eyes of 92 clinically diagnosed keratoconus patients enrolled in the Department of Ophthalmology of the Medical University of Graz (Graz, Austria) between 2008 and 2018 were reviewed. Approval of the institutional ethics committee was obtained and the study was conducted in accordance with the tenets of the Declaration of Helsinki.

Inclusion criteria were the diagnosis of manifest or subclinical keratoconus made by an experienced cornea specialist (G.S.) based on typical tomographic patterns. Exclusion criteria included corneal diseases other than keratoconus, extensive corneal scarring, corneal edema, and a history of ocular surgery such as corneal cross-linking, implantation of intracorneal rings or corneal refractive surgery.

For each patient, a Scheimpflug (Pentacam, Software version 6.09r40, Oculus Optikgeräte GmbH, Wetzlar, Germany) measurement was performed. Only images with a quality check status of “ok” were included. Analyzed locations to potentially describe the center of the keratoconus included: point of maximum axial curvature of the front surface (Kmax), point of maximum tangential curvature of the front surface (tKmax), point of minimum pachymetry (Pachymin), point of maximum elevation of the front surface (ELEF), and point of maximum elevation of the back surface (ELEB).

To calculate the distances between the described locations, cartesian coordinate system provided by the Pentacam System was used. The origin point of the coordinate system is the corneal vertex, the corneal intersect with the patients line of sight (12). While the coordinates of Kmax and Pachymin are automatically displayed by the Pentacam Software, the coordinates of tKmax, ELEF, and ELEB had to be determined manually by moving the computer mouse cursor to the desired position of the curvature or elevation map followed by extracting the displayed coordinates into a separate data file. After collecting coordinates of all locations, distances between points were calculated using the Pythagorean Theorem.

As a measure for keratoconus severity we used the D-value, a multimetric combination index composed of keratometric, pachymetric, pachymetric progression and elevation parameters which is provided by the Belin/Ambrosio Enhanced Ectasia Software of the Pentacam System.

Patient characteristics were reported as absolute and relative frequencies for categorical data and numerical data as mean and standard deviation. Correlations were determined using Spearman's correlation coefficient. For calculating correlations, only 1 randomized eye per patient was included. All statistical analyses were conducted using R version 4.1.2 (https://www.r-project.org).

## Results

**Figure 1:**
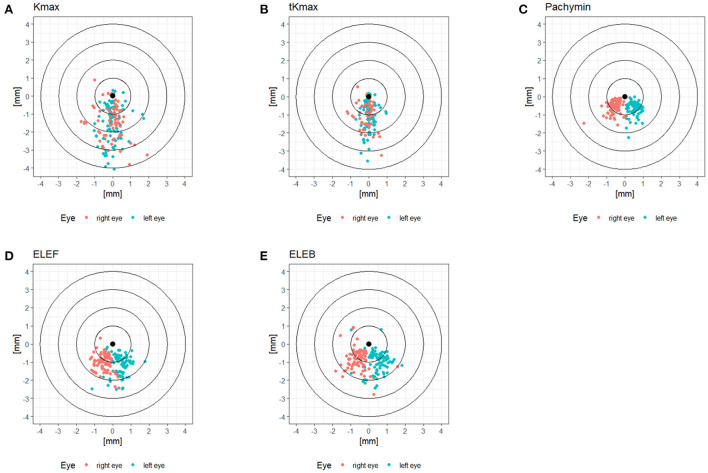
**(A–E)** Locations of tomographic parameters relative to the corneal vertex.

The patient characteristics are displayed in Table 1. The cohort comprised keratoconic eyes from subclinical to advanced stages, represented by a range of *D*-value from 1.0 to 20.5. The locations of Kmax, tKmax, Pachymin, ELEF, and ELEB relative to the corneal vertex are shown in Figure 1. While Pachymin, ELEF and ELEB were predominantly located in the inferior-nasal quadrant, the positions of Kmax and tKmax showed inferior clusters with a less pronounced lateral shifting.

**Table 1:** Patient characteristics.

**Parameter**	**Mean ±SD**
**Patients (*****n*** **=** **92)**
Age (years)	27.7 ± 7.8
**Eyes (*****n*** **=** **162)**
Right eyes	81 (49.7%)
*D*-value	7.8 ± 3.7
Kmax (D)	54.4 ± 6.1
Pachymin (μm)	471 ± 37

**Figure 2:**
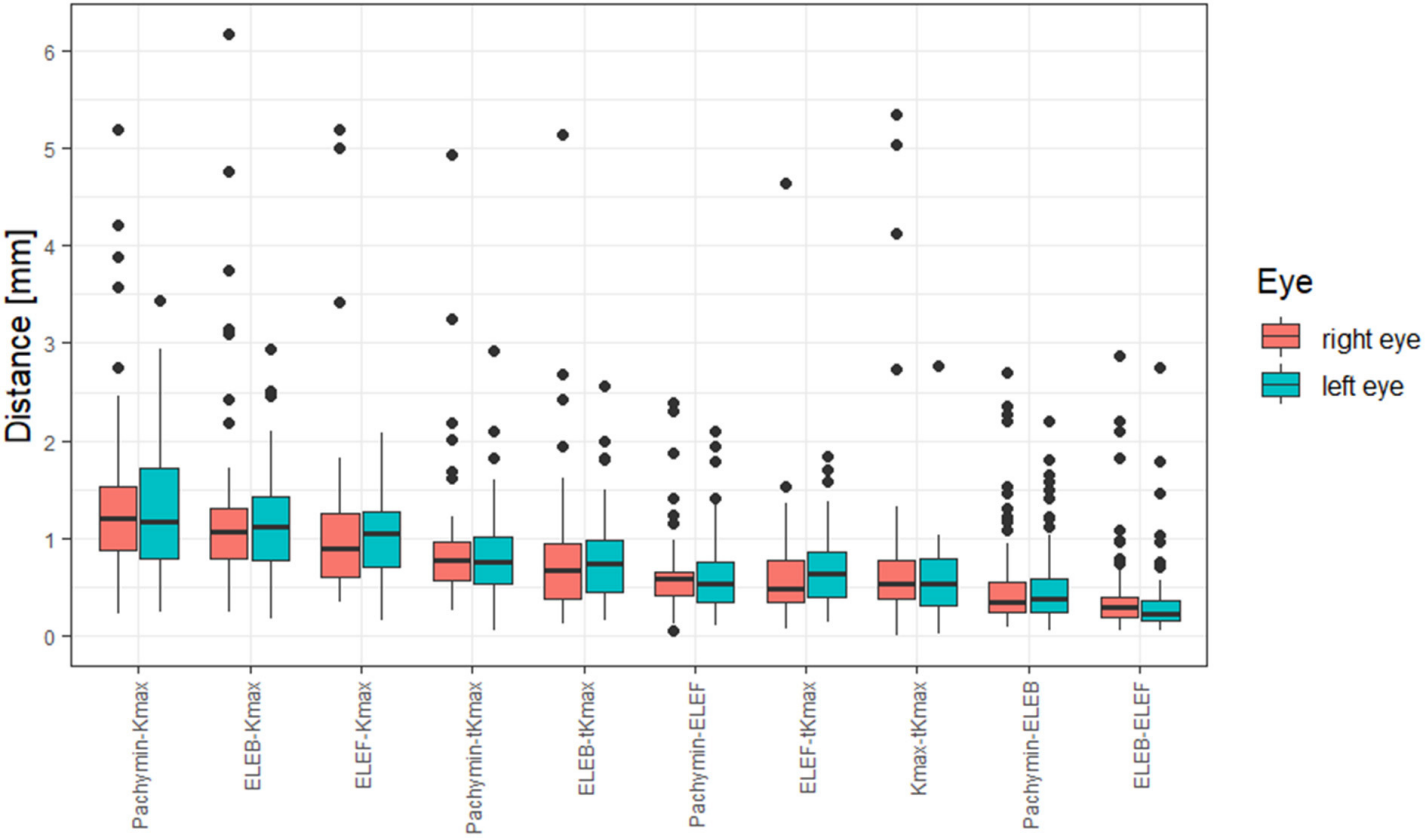
Box and whisker plot of distances between locations of different tomographic parameters.

Distances between the different points are shown in Table 2. The largest distances between locations were found between Kmax and Pachymin, Kmax and ELEB, and Kmax and ELEF (Figure 2). Thus, cone locations derived from the axial curvature map (Kmax) showed the lowest agreement with the locations determined by pachymetry or elevation maps. Low distances (<0.5 mm) were calculated between ELEB and ELEF, and ELEB and Pachymin.

**Table 2:** Distances between locations of different tomographic parameters.

**Characteristic**	**Overall, *N* = 162^a^**	**Right eye, *N* = 81^a^**	**Left eye, *N* = 81^a^**
Distance Pachymin-ELEB	0.37 (0.24, 0.58)	0.35 (0.25, 0.56)	0.38 (0.24, 0.59)
Distance Pachymin-ELEF	0.58 (0.40, 0.72)	0.58 (0.42, 0.65)	0.54 (0.34, 0.76)
Distance Pachymin-Kmax	1.19 (0.87, 1.60)	1.20 (0.88, 1.54)	1.17 (0.84, 1.73)
Distance Pachymin-tKmax	0.77 (0.55, 1.01)	0.78 (0.56, 0.97)	0.77 (0.53, 1.02)
Distance ELEB-ELEF	0.27 (0.17, 0.38)	0.29 (0.20, 0.41)	0.23 (0.16, 0.37)
Distance ELEB-Kmax	1.12 (0.79, 1.41)	1.07 (0.79, 1.32)	1.13 (0.77, 1.43)
Distance ELEB-tKmax	0.70 (0.41, 0.98)	0.67 (0.38, 0.95)	0.73 (0.45, 0.98)
Distance ELEF-Kmax	0.97 (0.64, 1.27)	0.89 (0.61, 1.26)	1.05 (0.69, 1.27)
Distance ELEF-tKmax	0.57 (0.36, 0.81)	0.49 (0.35, 0.78)	0.64 (0.39, 0.87)
Distance Kmax-tKmax	0.54 (0.35, 0.78)	0.54 (0.38, 0.77)	0.55 (0.32, 0.81)

^a^Median (IQR).

**Figure 3:**
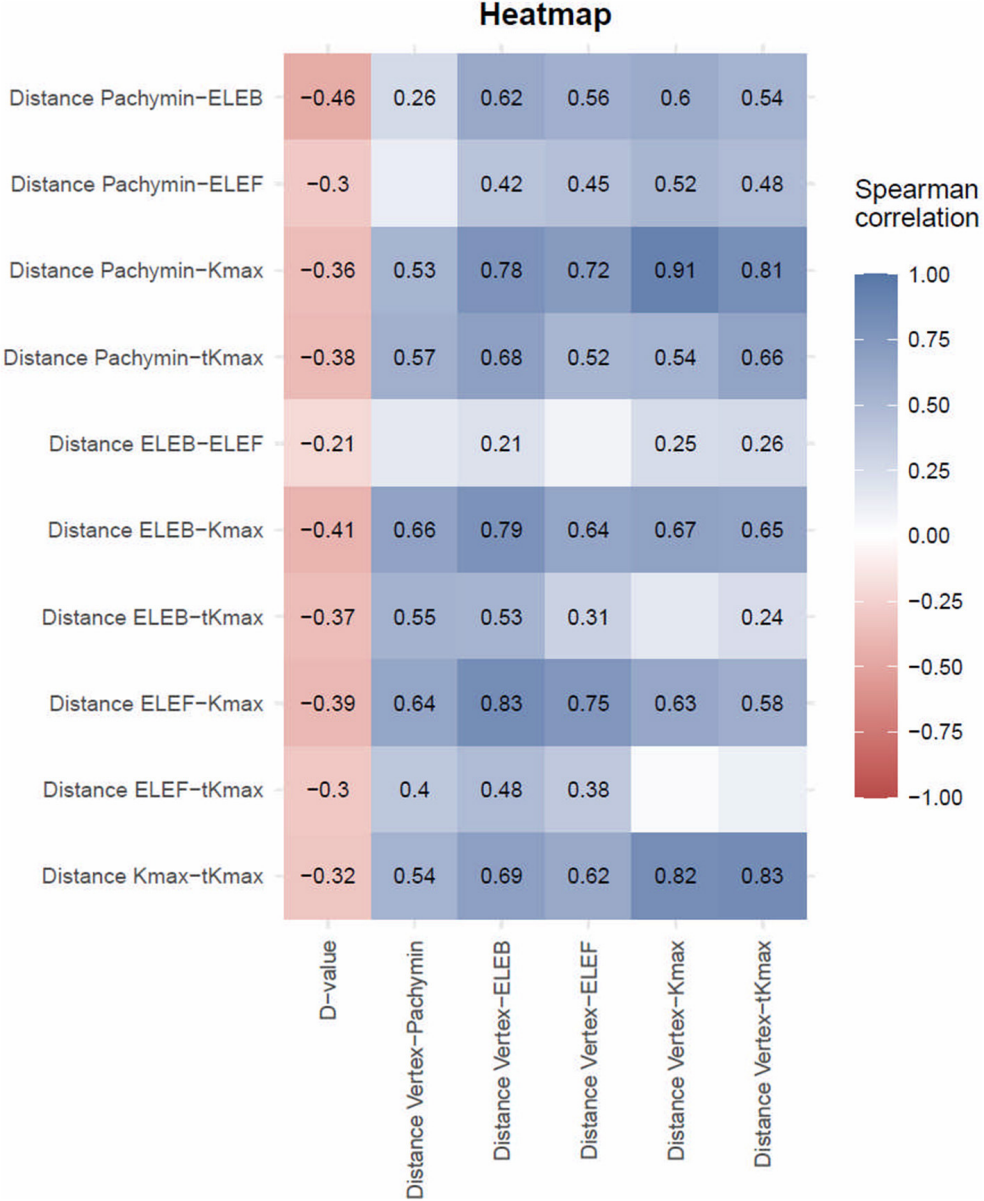
Spearman Correlation between distances between locations of different tomographic parameters and D-values or distances between locations and corneal vertex. The Spearman correlation coefficient is only presented in fields reaching statistical significance (*p* < 0.05). Discrepancy between different measures decreased with disease severity and increased with increasing distance from the corneal vertex.

Significant correlations (*p* < 0.05) were found between keratoconus severity and all of the calculated distances between the locations (Figure 3). Higher D-values and thus more advanced keratoconus showed a moderate correlation to smaller distances between ELEB and Kmax, ELEB and Pachymin, Kmax and Pachymin, and Kmax and tKmax. The majority of the distances between locations were positively correlated with the distance of those locations from the corneal vertex, meaning that more peripheral cones showed higher deviations between the locations. This observation was most pronounced for the distance between Kmax and Pachymin.

## Discussion

In our study, we analyzed deviations between different parameters which could be used to determine the center of a keratoconus and how they are impacted by disease severity. We revealed clinically significant inconsistencies of the location of the cone center when determined by different morphological maps, including axial or tangential curvature, pachymetry, or elevation maps.

The distribution patterns of the cone center relative to the corneal vertex differed between locations derived from axial or tangential curvature maps of the front surface (Kmax, tKmax) and locations based on pachymetry (Pachymin) or elevation data (ELEF, ELEB). While pachymetry and elevation data resulted in quite regular clusters in the inferior-nasal quadrant, the distribution of the curvature data showed more variability with less lateral shifting and only a moderate inferior predominance (Figure 1). These differences could be attributed to the masking effect of the corneal epithelium. Front stromal surface cones may be fully or partially masked by epithelial remodeling characterized by compensatory thinning over the stromal cone with a surrounding annulus of thicker epithelium (13). In early stages of keratoconus these effects may be great enough to mask stromal irregularities from curvature maps of the front surface, and in more advanced disease stages at least the location of the cone center could be obscured.

To quantify the discrepancies between locations of the cone center, we calculated the distances between locations for a pairwise comparison. The highest deviations were found between curvature-based locations (Kmax, tKmax) and pachymetry- or elevation-based locations (Pachymin, ELEF, ELEB). One reason for Kmax being the least consistent location may be that axial maps assume center points of surface curvature to be always located on the central reference axis hence reducing the sensitivity in identifying surface irregularities (14). Tangential curvature maps typically are more sensitive for describing focal corneal irregularities although they therefore also have higher noise-to-signal ratios. Hence, tKmax showed intermediate deviations in our cohort, better than Kmax, but less consistent than pachymetric- or elevation-based location data. The shortest distance and thus the highest consistency occurred between the elevation (ELEF, ELEB) and the pachymetry maps (Pachymin). As locations derived from the posterior corneal surface are less influenced by epithelial masking, we assume ELEB is the most accurate location for describing the center of a keratoconus. Showing only small deviations from ELEB, ELEF and Pachymin may be good alternatives for that purpose. Kmax and tKmax seem to be the least suitable options for clinical applications requiring the accurate center of the cone, as they had the least consistency with ELEB.

Our findings are in accordance with Sedaghat et al., who analyzed 90 keratoconic eyes and observed no considerable agreement between the elevation and axial curvature map in locating the center of the cone. Furthermore, similar to our study, the cone was found in the inferotemporal quadrant in the majority of cases (~95%) on the elevation map, while this quadrant contained only 18% of the cone center on the axial curvature map (15).

On the basis from the data of our study, we suggest that using axial or tangential curvature maps (Kmax and tKmax) is not a suitable option for locating a keratoconus. Clinical applications requiring the accurate center of the cone can be more efficient with elevation or pachymetry maps.

The pachymetry map is used for the Belin/Ambrosio enhanced best-fit sphere method, a popular method for the detection of early or subclinical keratoconus as well as for evaluating disease progression (16–18). With this method, the height of the cone is obtained by the difference in elevation between the best-fit sphere of the whole cornea and the best-fit sphere after excluding a fixed area around the thinnest point. Our study provides support for the reasonability to use pachymetry data for determining the cone location, as Pachymin showed only minor deviations from elevation-derived cone locations. Cunha et al. had a different view and decided to use Kmax for defining the keratoconus center in their recent study analyzing keratoconus enlargement as a predictor of keratoconus progression (19).

Another important application requiring a precise definition of the cone center is customized cross-linking, which is associated with more corneal flattening and a better visual outcome compared to conventional cross-linking. Seiler et al. (6) and Cassagne et al. (8) defined the keratoconus center based on the posterior elevation map, while Shetty et al. (10) decided to use axial and tangential curvature maps. A recent study from Lopes et al. confirmed remarkable variations in finding the center of a keratoconus with low agreement even in corneal specialists (20).

Our results showed evidence that with increased disease severity, the distances between the cone locations reduced. We suggest that with more pronounced ectatic alterations of the cornea the obscuring effects including epithelial masking and mathematical assumptions for curvature calculation diminish. On the other hand, special consideration should be given in defining the cone location during early stages of the disease, since larger differences between the locations can be expected. In contrast to our findings, Sedaghat et al. reported no considerable change of their results after re-analysis of data in different stages of keratoconus (15). Interestingly, in their investigation of 309 keratoconic patients Eliasy et al. demonstrated a reduction of the distance from the cone center to the corneal vertex with increased keratoconus severity. However, they did not analyze differences between cone locations obtained by different morphological maps (21).

When testing correlations, we detected a significant increase of deviations between the different locations with increasing distance from the corneal vertex. Thus, more peripheral cones showed higher discrepancies than more central cones. This observation was most pronounced for the distance between Kmax and Pachymin, which may be attributed to a relative central shift of the thinnest point of an ectatic cornea (Pachymin) due to the normal thickness profile of a human cornea, being thinnest centrally.

In conclusion, we found low consistency between different methods for describing the location of a keratoconus. As curvature-based determinations (like Kmax or tKmax) of the cone center showed the highest deviations, they should not be used for that purpose. Elevation- or pachymetry-based measures (like ELEB, ELEF, or Pachymin) are more suitable options for clinical applications requiring the accurate center of the cone. However, the discrepancies between the different cone location methods diminished with increasing disease severity and more central position of the cone.

## Data availability statement

The raw data supporting the conclusions of this article will be made available by the authors, without undue reservation.

## Ethics statement

The studies involving human participants were reviewed and approved by Ethics Committee of the Medical University of Graz. Written informed consent from the participants' legal guardian/next of kin was not required to participate in this study in accordance with the national legislation and the institutional requirements. Written informed consent was not obtained from the minor(s)' legal guardian/next of kin for the publication of any potentially identifiable images or data included in this article.

## Author contributions

GS, AK, and MSh contributed to conception and design of the study. AK and MSo organized the database. AB performed the statistical analysis and wrote sections of the manuscript. GS wrote the first draft of the manuscript with constructive inputs of JH-W, EL, NW, WL, and AW. All authors contributed to manuscript revision, read, and approved the submitted version.

## Conflict of interest

The authors declare that the research was conducted in the absence of any commercial or financial relationships that could be construed as a potential conflict of interest.

## Publisher's note

All claims expressed in this article are solely those of the authors and do not necessarily represent those of their affiliated organizations, or those of the publisher, the editors and the reviewers. Any product that may be evaluated in this article, or claim that may be made by its manufacturer, is not guaranteed or endorsed by the publisher.
